# Age-specific effects on the prognosis after surgery for gastric cancer: A SEER population-based analysis

**DOI:** 10.18632/oncotarget.9548

**Published:** 2016-05-21

**Authors:** Peng Song, Lei Wu, Bo Jiang, Zhijian Liu, Ke Cao, Wenxian Guan

**Affiliations:** ^1^ Department of General Surgery, Nanjing Drum Tower Hospital, The Affiliated Hospital of Nanjing University Medical School, Nanjing, China; ^2^ Department of Laboratory Medicine, The First Affiliated Hospital of Nanjing Medical University, Nanjing, China; ^3^ Department of Critical Care Medicine, Nanjing Drum Tower Hospital, The Affiliated Hospital of Nanjing University Medical School, Nanjing, China

**Keywords:** age, prognosis, gastric cancer, SEER

## Abstract

Prognosis of age at diagnosis for gastric cancer (GC) has been investigated in a few studies with inconclusive results. To assess the survival of GC across different age groups, we searched the Surveillance, Epidemiology, and End Results (SEER) database (1988-2010) and identified 10,092 patients undergoing gastrectomy. Analyses of the associations between age and 5-year GC-specific survival (GCSS) were carried out using the Kaplan-Meier method and Cox regression model. When the 50-59 year age group was used as reference group, patients younger than 50 years suffered similar survival rates, and the risk of death increased for patients older than 60 years (hazard ratio [HR], 1.11; 95% confidence interval [CI], 1.02-1.20), peaking for ages > 80 years (HR, 1.60; 95% CI, 1.46-1.76). Overall, HRs of 5-year GCSS increased steadily with age, even when age was evaluated as a continuous variable. We assessed the survival differences associated with age between three groups, using the cut-off ages of 30 and 50 years. Compared with the elderly group, a high survival rate was observed in the mid-age group, but not in the youngest group. Stratified analysis for sex, race, tumor site, histology and clinical stage yielded consistent results. This study shows that the prognosis of GC varies with age, and young GC patients appear to have a favorable GCSS after surgical treatment. Further studies are warranted to verify our findings.

## INTRODUCTION

Each year, almost one million new gastric cancer (GC) cases are diagnosed and seven hundred thousand patients die worldwide, establishing this disease as the fifth most common malignancy and the third leading cause of cancer related deaths [1]. GC mainly occurs in older populations, with a peak reported incidence for patients from 60 to 70 years [2]. Since the middle of the 20th century, the prevalence of GC has steadily decreased, but reverse trends are observed in the young generation [3–5]. Some studies have been conducted concerning the demographic characteristics, clinicopathological features and prognostic factors of GC in young patients. Young GC patients usually have advanced stage and undifferentiated tumors at presentation [6]. Because endoscopic screening has not been performed routinely among these groups, even in developed countries, diagnosis has been delayed, especially for the asymptomatic patients [7]. Besides, GC in young patients seems to spread more rapidly and reveals a more biologically aggressive form [8].

Knowledge of the important prognostic factors in the development of GC could help us understand this disease and make crucial therapeutic measures. Age at diagnosis, a key variable, not only is used as an indispensable adjusted element in the observational studies, but also contains inestimable value for prognosis. In recent years, several studies have investigated the prognostic outcome of GC in young patients compared with the elderly, but yield inconclusive results. It has been suggested that young patients suffer worse survival rates due to patient characteristics and varied tumor behavior [7, 9]. Despite these unfavorable conditions for young patients, some researchers argue that their survival rates are better, at least equivalent to the elderly [10–12]. In this study, population-based data from the National Cancer Institute's Surveillance, Epidemiology and End Results (SEER) program has been used to evaluate age-specific effects on the prognosis of GC.

## RESULTS

### Characteristics and clinical features of patients

In this study, 10,092 patients were identified who have been diagnosed with GC and fulfill the inclusion criteria stated in METHODS. Considering the long time span and different staging schemas in this study, we divided SEER dataset into two time periods (1988-2003 and 2004-2010). The demographic characteristics and clinical features of these patients are shown in Table 1. The median follow-up times were 25 and 30 months for the study periods 1988-2003 and 2004-2010, respectively. In a follow-up period of 299 months, a total of 5,820 patients died as a direct result of GC. There were 3,077 males (61.0%) and 1,970 females (39.0%) in the study period 1988-2003, and study period 2004-2010 included 3,155 (62.5%) males and 1,890 (37.5%) females. Higher percentages of white race, black race, cardia type, intestinal type, stage I and stage II were observed in the period 2004-2010 compared with those in the period 1988-2003.

**Table 1:** Characteristics and clinical features of gastric cancer patients

	Study Period 1988-2003				Study Period 2004-2010				
Variable	Patients *n*= 5047		Deaths *n*= 3238		Patients *n*= 5045		Deaths *n*= 2582		*P*^a^
	No.	%	No.	%	No.	%	No.	%	
**Follow-up** (months)	Median 25, IQR 11-109				Median 30, IQR 14-54				
**Age** (years)									
< 30	30	0.6	19	0.6	30	0.6	11	0.4	< 0.001
30-39	180	3.6	100	3.1	147	2.9	58	2.3	
40-49	508	10.1	282	8.7	507	10.1	230	8.9	
50-59	898	17.8	561	17.3	1046	20.7	467	18.1	
60-69	1354	26.8	865	26.7	1356	26.9	664	25.7	
70-79	1488	29.5	990	30.6	1296	25.7	718	27.8	
≥ 80	589	11.7	421	13.0	663	13.1	434	16.8	
**Sex**									
Female	1970	39.0	1179	36.4	1890	37.5	957	37.1	0.105
Male	3077	61.0	2059	63.6	3155	62.5	1625	62.9	
**Race**									
White	3050	60.4	2062	63.7	3230	64.0	1732	67.1	< 0.001
Black	584	11.6	370	11.4	611	12.1	330	12.8	
Other^b^	1413	28.0	806	24.9	1204	23.9	520	20.1	
**Tumor sites**									
Cardia	1206	23.9	886	27.4	1395	27.7	770	29.8	< 0.001
Non-cardia	2956	58.6	1718	53.1	2879	57.1	1355	52.5	
Other^c^	885	17.5	634	19.6	771	15.3	457	17.7	
**Histology**									
Intestinal	927	18.4	558	17.2	1211	24.0	531	20.6	< 0.001
Diffuse	1474	29.2	997	30.8	1491	29.6	853	33.0	
Unknown	2646	52.4	1683	52.0	2343	46.4	1198	46.4	
**TNM stage**									
I	829	16.4	211	6.5	1132	22.4	199	7.7	< 0.001
II	1187	23.5	551	17.0	1301	25.8	519	20.1	
III	3031	60.1	2476	76.5	2612	51.8	1864	72.2	

a Two-sided χ^2^ test for the frequency distributions of patients between the period 1988-2003 and 2004-2010.

b Including American Indian, Alaska Native, Asian, Pacific Islander and Unknown.

c Including Site overlapping, Unspecified and Unknown.

IQR: interquartile range (from 75^th^ percentile to the 25^th^ percentile).

**Figure 1:**
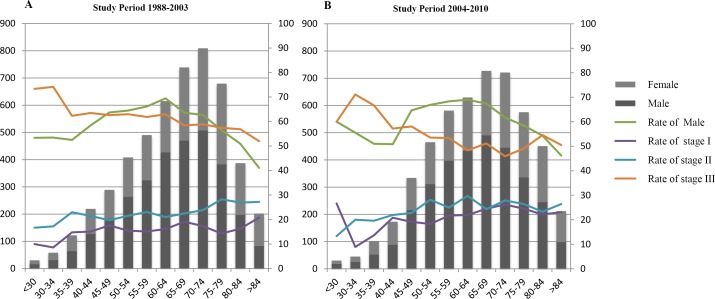
Distribution of sex and TNM stage among age groups for two study periods **A.** Study period 1988-2003, **B.** Study period 2004-2010. The primary vertical axis on the left refers to patient number, whereas the secondary vertical axis on the right side refers to the proportion of patients within various categories.

Figure 1 displays the distribution of gender frequency and clinical stage across different age groups in two study periods. The rate of male GC diagnosis was consistently higher than that of female diagnosis except in the group of age > 84 years. The proportion of patients with stage III GC presented a downward trend overall.

### Impact of age on GC survival outcomes for two cohorts

**Figure 2:**
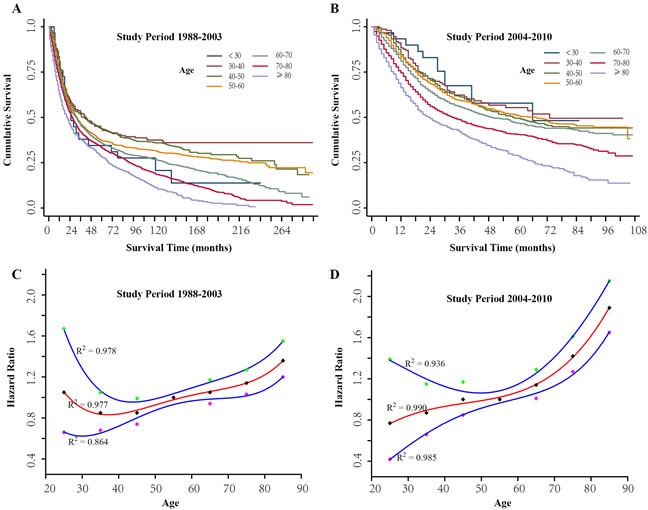
The prognosis of gastric cancer patients for different age groups **A.** Kaplan-Meier estimates of gastric cancer-specific survival (GCSS) in different age groups for study period 1988-2003. **B.** Kaplan-Meier estimates of GCSS in different age groups for study period 2004-2010. **C.** Estimates of hazard ratios (HRs) of 5-year GCSS changing with age using quartic polynomial regression for study period 1988-2003. **D.** Estimates of HRs of 5-year GCSS changing with age using quartic polynomial regression for study period 2004-2010. The solid red lines represent HR estimates, and the solid blue lines represent 95% confidence intervals. R2 represent the coefficient of determination.

We focused on the age-related differences in GC-specific survival (GCSS) for two cohorts (study period 1988-2003 and study period 2004-2010). Kaplan-Meier plots show that patients who were 30-50 years of age at diagnosis had the best survival rate in all the subgroups, and those younger than 30 years and older than 80 years presented the worst survival rates in the study period from 1988 to 2003 (Figure 2A). In the period 2004-2010 patients younger than 30 years had the best prognosis, and those older than 80 years still exhibited the worst survival rates (Figure 2B). Considering the difference in follow-up time, the Cox regression model was employed to further evaluate the effect of age on 5-year GCSS. We used the 50- to 59-year-old group as the reference for univariate analyses based on the Kaplan-Meier results. As shown in Figure 2C, for study period 1988-2003, the hazard ratio (HR) of 5-year GCSS increased with age, from 0.85 [95% confidence interval (CI), 0.68-1.05] in the group aged 30-39 years to 1.36 (95% CI, 1.20-1.55) in the group older than 80 years, while the patients younger than 30 years did not have better survival rates (HR, 1.05; 95 % CI, 0.66-1.67). For the study period 2004-2010, the HR of 5-year GCSS gradually increased with age, from 0.77 (95% CI, 0.42-1.39) in the group younger than 30 years to 1.89 (95%, 1.65-2.15) for the group older than 80 years (Figure 2D). We developed a quartic polynomial regression which was fitted to reflect the correlation between change in HR and age.

To control potential confounders, stratified analyses were performed by key study characteristics and clinical factors (Supplementary Table 1 and Supplementary Table 2). In both periods of diagnosis 1998-2003 and 2004-2010, younger groups (age 30-39 and 40-49 years) had a favorable prognosis, while older groups (age 60-69, 70-79, ≥ 80 years) exhibited a worse survival rate. Significant associations were observed among subgroups of patients who were male, white, exhibited cardia GC, and stage I GC for age 40-49 years group, and patients who were male and exhibited non-cardia, diffuse, stage I GC for age 70-79 years group, all strata for age ≥ 80 years age group in the period 1998-2003; and of non-cardia, stage I, stage III for age 60-69 group, all strata for age 70-79 and ≥ 80 years groups in the period 2004-2010. The age < 30 years groups exhibited different outcomes with no statistically significant difference for the study periods 1998-2003 and 2004-2010, respectively.

### Impact of age on GC survival outcomes for entire cohort

**Figure 3:**
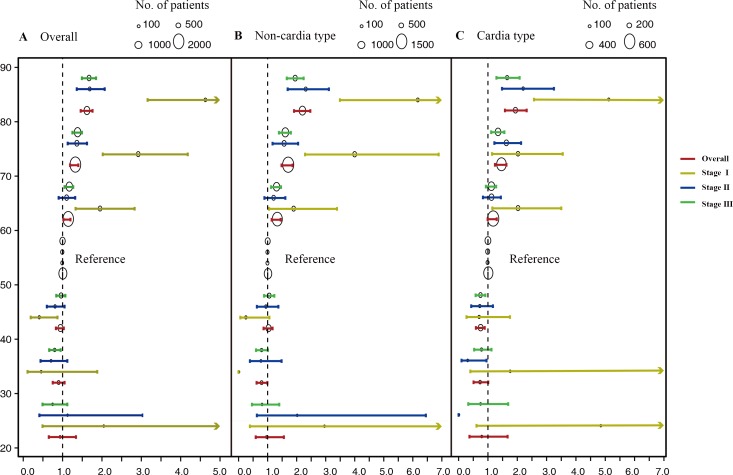
Estimates of hazard ratios (HRs) of 5-year gastric cancer-specific survival changing among different age groups for entire cohort under different TNM stage **A.** The overall group, **B.** The non-cardia cancer type group. **C.** The cardia cancer type group. The circles represent the number of patients.

As age had a similar effect on the prognosis in the two study cohorts, we then merged the groups for further analysis of the 5-year GCSS. In entire cohort, sex, race, histology, and especially tumor sites violated proportional hazards (PHs) assumption indicated by Therneau-Grambsch tests with rho (ρ) and chi-square (χ^2^) values. When focus was confined to the non-cardia and cardia type, we found these variables were satisfied with PHs assumption (Supplementary Table 3). Therefore, we applied univariate Cox regression for the overall survival analysis and multivariate Cox regression for the stratified analysis of non-cardia and cardia. As illustrated in Table 2, compared to the referent age group of 50 to 59 years, the age < 30 years group, 30-39 years group and 40-49 years groups had better survival rates whereas statistically significant differences were only observed in the age 30-39 years group with non-cardia type (HR 0.78; 95% CI, 0.62-0.99) and age 40-49 years group with cardia type (HR 0.73; 95% CI, 0.59-0.89), and age 60-69, 70-79 and ≥ 80 groups had significantly different HRs for survival. We also evaluated the effects of age on the prognosis under different TNM stages. Figure 3 displays the interaction effect of TNM stage and age: the older a patient, the worse the prognosis associated with stage I compared with stage II and stage III. For stage I, the HRs of GCSS plotted against the different age groups seemed to form a V-shaped curve, and patients younger than 40 years did not have a favorable prognosis, and patients older than 60 years had worse survival rates.

**Table 2:** Analysis of gastric cancer-specific survival across different age groups

	Overall		Non-cardia		Cardia	
**Age** (years)	Death/Patients (%)	HR (95% CI)^a^	Death/Patients (%)	HR (95% CI)^b^	Death/Patients (%)	HR (95% CI)^b^
< 30	30/60 (50.0)	0.93 (0.65-1.34)	17/35 (48.6)	0.95 (0.59-1.56)	7/11 (63.6)	0.78 (0.37-1.67)
30-39	158/327 (48.3)	0.89 (0.75-1.05)	81/188 (43.1)	**0.78 (0.62-0.99)**	37/73 (50.9)	0.72 (0.51-1.01)
40-49	512/1015 (50.4)	0.93 (0.83-1.03)	273/591 (46.2)	1.01 (0.86-1.17)	132/249 (53.0)	**0.73 (0.59-0.89)**
50-59	1028/1944 (52.9)	Reference	456/1024 (44.5)	reference	382/620 (61.6)	reference
60-69	1529/2710 (56.4)	**1.11 (1.02-1.20)**	745/1476 (50.5)	**1.29 (1.14-1.45)**	499/796 (62.7)	1.13 (0.99-1.30)
70-79	1708/2784 (61.4)	**1.29 (1.19-1.40)**	956/1675 (57.1)	**1.66 (1.48-1.86)**	443/652 (67.9)	**1.42 (1.24-1.63)**
≥ 80	855/1252 (68.3)	**1.60 (1.46-1.76)**	545/846 (64.4)	**2.17 (1.91-2.47)**	156/200 (78.0)	**1.92 (1.58-2.32)**

a Univariate Cox regression

b Multivariate Cox regression, adjusted for sex, race, histology and TNM stage.

HR, hazard ratio; CI, confidence interval.

**Figure 4:**
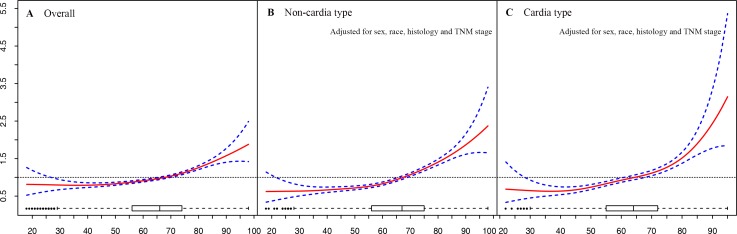
Relationship between age and hazard ratio of 5-year gastric cancer-specific survival **A.** overall group, **B.** non-cardia cancer type group. **C.** cardia cancer type group. The hazard ratio is related to an unspecified baseline hazard function for the reference with all covariates equal to 0. Box plot at the bottom shows the distribution of age.

**Figure 5:**
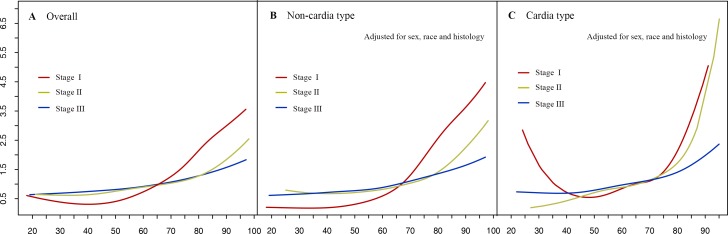
Relationship between age and hazard ratios of 5-year gastric cancer-specific survival under different TNM stage **A.** Overall group, **B.** Non-cardia cancer type group. **C.** Cardia cancer type group. The hazard ratio is related to an unspecified baseline hazard function for the reference with all covariates equal to 0.

We then examined age as a continuous variable to reveal its association relative to 5-year GCSS. Therneau-Grambsch tests show age is a time-dependent covariate, so extending the Cox-regression model was performed using penalized smoothing splines. The univariate Cox analysis for the entire cohort indicates that as the age increased, the risk of death for GC demonstrated an increase in a dose-response manner, with no effect of age up to about 30 years old, followed by a sharp increase at about 70 years old (Figure 4A). Similar straightforward dose-effect relationships were also observed in non-cardia and cardia cancer types (Figure 4B-4C). In the stratified analysis of TNM stage, the age-specific effects were more prominent in stage I than in stage II and stage III (Figure 5). Thereafter, we created an interaction term to determine the associations of age and different TNM stages in predicting GCSS. Significant interactions between age and TNM stage were found in the multivariate analysis for non-cardia and cardia GC, respectively (both *P* < 0.001).

### Comparison of survival between the three groups

**Figure 6:**
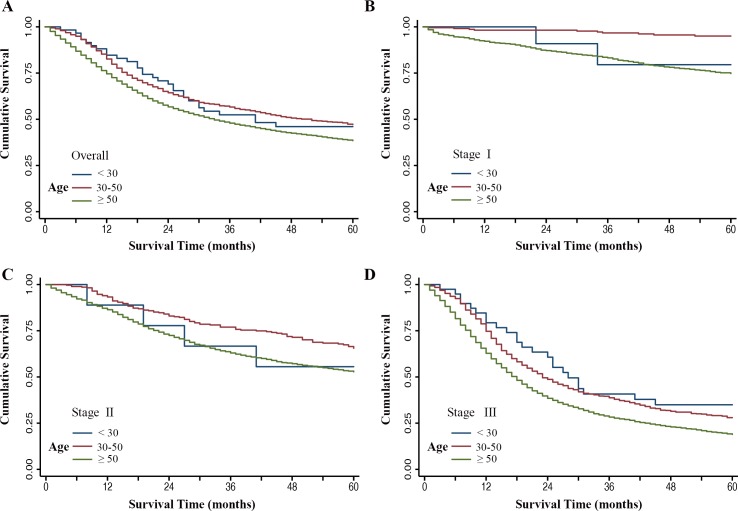
Kaplan-Meier estimates of gastric cancer-specific survival among three groups (age < 30 years group, age 30-50 years group, age ≥ 50 years group) **A.** The overall group, **B.** Stage I group, **C.** Stage II group, **D.** Stage III group.

Based on the aforementioned results, we divided the total study set into three groups: extremely young group (age < 30 years), young group (30-50 years), and elderly group (≥ 50 years), to further investigate the survival differences associated with age. The extremely young group and young group contained a higher proportion of female patients than the elderly group (43.3% *vs*. 41.3% *vs*. 37.7%). Besides, significant differences were found in race (*P* < 0.001), histology (*P* < 0.001), and TNM stage (*P* < 0.001) among the three groups (Table 3). Diffuse type (55.0% *vs*. 48.4% *vs*. 26.3%) and stage III (66.7% *vs*. 62.0% *vs*. 54.9%) were observed more frequently either in the extremely young group or young group compared with elderly group. Patients in young group were associated with an obviously favorable survival than elderly patients, but not in the extremely young group (Figure 6A). In the stratified analysis, survival was significantly decreased in young patients in the subgroups of both female and male, white and black race, cardia and non-cardia, intestinal and diffuse type (Table 3). Moreover, it is worth mentioning that the protective effect associated with age was found in each TNM stage for the young group (stage I, Figure 6B; stage II, Figure 6C; and stage III, Figure 6D; all log-rank *P* < 0.001), and stage III for extremely young group (Figure 6D, log-rank *P* = 0.015), compared to the elderly group.

**Table 3:** Stratified analysis of age at diagnosis associated with gastric cancer patients' survival

	Extremely young group		Young group		Elderly group	
Variable	Death/Patients (%)	HR (95% CI)^a^	Death/Patients (%)	HR (95% CI)^a^	Death/Patients (%)	*P*^b^
**Overall**	30/60 (50.0)	0.78 (0.54-1.11)	670/1342 (49.9)	**0.77 (0.71-0.83)**	5120/8690 (58.9)	
**Sex**						0.033
Female	13/26 (50.0)	0.84 (0.48-1.45)	280/554 (50.5)	**0.83 (0.73-0.94)**	1843/3280 (56.2)	
Male	17/34 (50.0)	0.74 (0.46-1.19)	390/788 (49.5)	**0.73 (0.66-0.81)**	3277/5410 (60.6)	
**Race**						< 0.001
White	18/38 (30.0)	0.67 (0.42-1.06)	430/805 (53.4)	**0.79 (0.72-0.88)**	3346/5437 (61.5)	
Black	3/5 (60.0)	1.26 (0.40-3.98)	115/225 (51.1)	**0.74 (0.61-0.91)**	582/965 (60.3)	
Other^c^	9/17 (52.9)	0.98 (0.51-1.89)	125/312 (40.1)	**0.69 (0.57-0.83)**	1192/2288 (52.1)	
**Tumor sites**						0.113
Cardia	7/11 (63.6)	0.65 (0.31-1.38)^e^	169/322 (52.5)	**0.60 (0.51-0.70)**^e^	1480/2268 (65.3)	
Non-cardia	17/35 (48.6)	0.67 (0.41-1.09)^e^	354/779 (45.4)	**0.67 (0.60-0.75)**^e^	2802/5021 (53.8)	
Other^d^	6/14 (42.9)	0.53 (0.24-1.19)	147/241 (61.0)	**0.84 (0.71-1.01)**	938/1401 (67.0)	
**Histology**						< 0.001
Intestinal	4/7 (57.1)	1.04 (0.39-2.80)	67/160 (41.9)	**0.74 (0.58-0.95)**	1018/1971 (51.7)	
Diffuse	17/33 (51.5)	0.64 (0.39-1.03)	349/649 (53.8)	**0.70 (0.62-0.79)**	1484/2283 (65.0)	
Other	9/20 (45.0)	0.76 (0.40-1.48)	254/533 (47.7)	**0.73 (0.64-0.83)**	2618/4436 (59.0)	
**TNM stage**						< 0.001
I	2/11 (18.2)	0.85 (0.21-3.42)	10/225 (4.4)	**0.17 (0.09-0.32)**	398/1725 (23.1)	
II	4/9 (44.4)	0.91 (0.34-2.44)	90/285 (31.6)	**0.63 (0.51-0.78)**	976/2194 (44.5)	
III	24/40 (60.0)	**0.60 (0.40-0.91)**	570/832 (68.1)	**0.74 (0.67-0.81)**	3746/4771 (78.5)	

a Univariate Cox regression, using the elderly group as referent.

b Two-sided χ^2^ test for the frequency distributions of patients among the three groups.

c Including American Indian, Alaska Native, Asian, Pacific Islander and Unknown.

d Including Site overlapping, Unspecified and Unknown.

e Multivariate Cox regression, adjusted for sex, race, histology and TNM stage.

HR, hazard ratio; CI, confidence interval.

## DISCUSSION

In the present study, we aimed to elaborate the age effects for survival among GC patients. Based on the SEER database which had the broad geographic coverage, a total of 10,092 patients were included to evaluate this impact. The lowest HR of 5-year GCSS was observed in patients who were diagnosed near the age of 30 years and the risk increased with age, being the highest for patients older than 80 years, whereas patients of extremely young age (< 30 years) did not have satisfactory prognosis. Overall, the older age at diagnosis correlates with a worse prognosis.

The female/male ratio, diffuse type and stage III were predominant in young GC patients. These results are consistent with those of previous studies. Gender difference may reflect the influence of sex hormones such as estrogens in the etiology of GC. Briefly, GC risk increases with early menopause and short duration of fertility, indicating that estrogens may have protective effect in the process of gastric carcinogenesis [13, 14]. One possible mechanism of estrogen-mediated prevention is through the reduction of gastric acid [15]. Lindblad and his colleagues proved the protective influence in a cohort of men heavily exposed to estrogens [16]. Gan, *et al*. immunohistologically investigated estrogen receptors in 866 GC patients and found that the positive expression is correlated with high tumor grade and intestinal type, and early TNM stage [17]. Matsui, *et al*. reported that the estrogen receptor positive rate is slightly higher in young females, and the prognosis of GC patients who are receptor-positive is still controversial [18]. This study showed a better survival for the young in both female and male population stratifying by sex. Using female GC patients as a reference group, the male group exhibited significantly worse survival (HR = 1.10, 95% CI = 1.03-1.56). However, restricted to young groups (age < 30 and 30-50 years old), risk of death for males was not significantly different from that of females (HR = 0.98, 95% CI = 0.48-2.04 for age < 30 years; HR = 0.98, 95% CI = 0.84-1.14 for age 30-50 years). Therefore, we hypothesized that age and sex hormones have an impact on the prognosis, especially the dominant role of age, but additional studies are warranted.

Despite diffuse type and advanced stage being prevalent in young patients and thereby a biologically more aggressive form of GC being present, the prognosis is still beneficial to the young. Even in the subgroups of diffuse type and each TNM stage, young age still was a favorable prognostic factor. On one hand, because all patients underwent surgical treatment, young patients could have advantageous physical conditions that conferred better tolerance to surgery and potentially fewer complications (eg. anastomotic leak, prolonged inflammation) and a quicker return of gastrointestinal function. The incidence of postoperative complications had an obvious impact on the overall survival of patients with GC even if the tumor was resected curatively [19, 20]. On the other hand, the worse survival of the elderly could be explained partly by inadequate treatment. As elderly patients were less likely to be enrolled in randomized trials and to receive comprehensive and standardized treatment, they might not gain a survival benefit from adjuvant chemotherapy or radiotherapy [21]. Hoffman, *et al*. reported that receipt of postoperative chemoradiation therapy did not significantly increase survival for elderly patients with resected gastric adenocarcinoma [22]. It is therefore valuable to conduct randomized trials that include more elderly GC patients to evaluate the impact of treatment regimens for this population.

For gastric malignant tumors, there is no universally accepted standard of the minimum number of lymph nodes examined, and removal of at least 15 lymph nodes at the time of gastrectomy has been recommended by the National Comprehensive Cancer Network (NCCN), except for the N0 patients. According to the previous studies as well as SEER data, the total number of retrieved lymph nodes is significantly correlated with prognosis [23, 24]. Two main reasons for dissecting a recommended number of lymph nodes are that this helps to standardize the surgical procedure and discover more metastatic lymph nodes. So the total lymph node number and the number of negative lymph nodes play important roles in the prognostic evaluation and treatment decisions [25, 26]. Some scholars advocated harvesting a large number of lymph nodes (more than 30) in radical gastrectomy [27]. In this study, the numbers of patients with lymph node metastasis in extremely young and young subgroups were higher than those in the elderly group [46/60 (76.7%) *vs*. 982/1342 (73.2%) *vs*. 6023/8690 (69.3%), *P* = 0.008]. Interestingly, the proportions of patients with more than 30 lymph nodes harvested were dramatically different among these three groups [(11/60 (18.3%) *vs*. 334/1342 (24.9%) *vs*. 1910/8690 (22.0%), *P* = 0.044]. This phenomenon could explain in part why even young patients with aggressive tumor characteristics, exhibited better survival rates, which contributed to the sufficient lymphadenectomy.

An important strength of our study was that the number of total subjects (10,092) was substantial and the follow-up time was adequate, which ensured relative reliability of our results. We focused on the GC patients with surgical opportunity, and excluded patients with carcinoma *in situ* and distant metastasis that had a great effect on the prognosis. GCSS was investigated in a wide age range, and the age was evaluated as a continuous variable, allowing us to be aware of the survival in different age classes. These elements made our study very different from previous research studies [28, 29].

Although this was a large population-based study, the following limitations apply. First, standardized administration of adjuvant chemotherapy, neoadjuvant chemotherapy and the duration of chemotherapy could contribute to a favorable prognosis. Due to the SEER database lacking this information, we could not analyze the impact of chemotherapy on survival in depth. It is quite possible that the treatment strategy and quality of surgical technology differed significantly from 1988 to 2010, but we found a similar trend towards better prognosis for younger patients in both subgroups of 1988-2003 and 2004-2010. Second, surgery was performed on each patient, but it was not equivalent to curative resection for resected GC, because we did not know specific surgical procedures and the extent of lymph node dissection. Third, young age was a protective factor not only in non-cardia GC, but a similar trend was observed in cardia subgroups. We should treat this finding regarding cardia GC with caution. Because of anatomic distinction of the esophagogastric junction in close proximity to either the gastric or esophageal carcinoma is ambiguous. The SEER registry currently does not provide sufficient data to distinguish the two. Fourth, there were only 60 patients in the age < 30 years group, and the small number of patients made this result uncertain for this unique community and the potential sources of bias were inevitable. Fifth, the existence of co-morbidities (i.e. general health status and nutritional status) are harmful factors for elderly patients, and these potential confounding factors could not be adjusted in our analyses due to limited data.

In conclusion, our results indicate that the prognosis of GC varies with age. Young patients suffer a higher survival rate after surgery compared to elderly patients, and GCSS becomes worse with increasing age. More well-designed studies are required to further clarify this association.

## MATERIALS AND METHODS

### Study population

Case listing session was obtained from the SEER program using SEER*Stat 8.2.1 (http://seer.cancer.gov/seerstat) [30]. The current SEER project includes 17 population-based cancer registries which represent about 28% of the US population. The SEER data are available to the public for the purpose of studying cancer-based epidemiology.

Patients diagnosed with GC during the period 1988 to 2010 were extracted from the SEER database according to the International Statistical Classification of Diseases, 10th Revision (ICD-10, site codes 16.0-16.9). Only patients at least 18 years of age with a diagnosis of gastric adenocarcinoma (ICD for Oncology, 3rd Edition [ICD-O-3] code in the range of 8140-8145, 8210-8211, 8221, 8255, 8260-8263, 8310, 8323, 8480-8481, 8490), and undergoing gastrectomy with at least 15 lymph nodes resected were eligible for this study. Exclusion criteria included carcinoma *in situ*, with distant metastasis (M1), inadequate or discordant staging information, and death within 30 days of surgery. The extent of disease codes (EOD10, 1988-2003) and collaborative staging (CS­­­­­, a unified data collection system, 2004+) were used to restage TNM classification in terms of the American Joint Committee on Cancer (AJCC) Cancer Staging Manual (7th edition, 2010). We restricted eligibility to adults in 1988 or later, because accurate staging were available to be performed since then. Patients diagnosed after 2010 were excluded to ensure adequate follow-up time. Age, sex, race, year of diagnosis, tumor sites, tumor grade, regional lymph node examination, TNM and GCSS were assessed in our study. Survival time was calculated from the date of diagnosis to the date of cancer-specific death. Patients alive on the last follow-up date or deaths attributed to causes other than GC were considered to be censored observations. When focusing on non-cardia GC (ICD-10 codes, 16.1-16.6), cases in cardia (ICD-10 code, 16.0), overlapping (ICD-10 code, 16.8), and unspecified subsites (ICD-10 code, 16.9) were not included. The definition of histology was based on ICD-O-3. The codes for intestinal subtype of GC were 8143-8144, 8210-8211, 8221, 8255, 8260-8263, 8310, 8323, 8480-8481. Diffuse subtype referred to codes 8141-8142, 8145 and 8490 [3].

### Statistical analysis

To clarify the impact of age at diagnosis on GCSS, we first classified age as a categorical variable of seven groups: younger than 30 years, 30-39 years, 40-49 years, 50-59 years, 60-69 years, 70-79 years, and older than 80 years. The group of patients aged 50-59 years was used as reference. Then we examined age as a continuous variable, and divided the age into triple classification (< 30 years, 30-50 years, and ≥ 50 years) to further determine age-specific effects on GCSS. Pearson's χ^2^ test was used for categorical variables to examine differences between select groups. Kaplan-Meier method with log-rank test was employed to evaluate survival curves. HRs and 95% CIs were estimated by Cox regression analysis. Potential nonlinear association between age and the HRs of GCSS was assessed using polynomial regression. Therneau-Grambsch tests discerning a correlation between scaled Schoenfeld residuals and time were used to test PH [31]. All the statistical analyses were done with SAS 9.1 and R 3.13. All *P* values were two-sided, and lower than 0.05 was considered statistically significant.

## SUPPLEMENTARY MATERIAL AND FIGURES


