# Supplementary material for: Age-specific effects on the prognosis after surgery for gastric cancer: A SEER population-based analysis
Source: Oncotarget. 2016 May 21;7(30):48614–24. doi: 10.18632/oncotarget.9548 (PMC5217043; doi:10.18632/oncotarget.9548)
Supplement: Supplementary file 1 [file oncotarget-07-48614-s001.pdf]

Age-specific effects on the prognosis after surgery for gastric cancer: A SEER population-based analysis

Supplementary Material

Supplementary Table 1: Stratified analysis of age at diagnosis associated with gastric cancer patients' survival in the period 1988-2003.

	**Age < 30 years**		**Age 30-39 years**		**Age 40-49 years**		**Age 50-59 years**		**Age 60-69 years**		**Age 70-79 years**		**Age≥ 80 years**	
Variable	Deaths /Patients	HR (95% CI)	Deaths /Patients	HR (95% CI)	Deaths /Patients	HR (95% CI)	Deaths /Patients	HR (95% CI)	Deaths /Patients	HR (95% CI)	Deaths /Patients	HR (95% CI)	Deaths /Patients	HR (95% CI)
**Sex**														
Female	8	1.12	48	1.01	102	0.9	177	1.00	273	1.1	365	1.15	206	**1.42**
	/14	0.55-2.29	/85	0.73-1.4	/196	0.71-1.15	/311	Reference	/457	0.91-1.34	/598	0.96-1.38	/309	**1.16-1.74**
Male	11	1.05	52	0.77	180	**0.83**	384	1.00	592	1.02	625	**1.16**	215	**1.43**
	/16	0.57-1.92	/95	0.57-1.02	/312	**0.7-1.00**	/587	Reference	/897	0.90-1.16	/890	**1.02-1.32**	/280	**1.2-1.69**
**Race**														
White	9	0.94	61	0.85	176	**0.84**	367	1.00	579	1.04	607	1.07	263	**1.28**
	/14	0.48-1.83	/102	0.65-1.12	/298	**0.7-1.00**	/538	Reference	/849	0.91-1.19	/890	0.94-1.22	/359	**1.09-1.51**
Black	2	1.65-	17	0.78	48	0.82	71	1.00	87	1.08	107	1.26	38	**1.59**
	/3	0.4-6.88	/33	0.46-1.33	/89	0.57-1.19	/119	Reference	/136	0.79-1.48	/152	0.93-1.71	/52	**1.07-2.37**
Other	8	1.28	22	0.91	58	0.91	123	1.00	199	1.06	276	**1.31**	120	**1.55**
	/13	0.62-2.64	/45	0.58-1.44	/121	0.67-1.25	/241	Reference	/369	0.84-1.33	/446	**1.06-1.63**	/178	**1.2-1.99**
**Tumor sites**														
Cardia	5	1.35	21	0.95	75	**0.74**	202	1.00	266	1.00	234	1.17	83	**1.52**
	/5	0.55-3.33	/32	0.6-1.49	/124	**0.56-0.96**	/272	Reference	/367	0.83-1.2	/305	0.97-1.42	/101	**1.17-1.97**
Non-cardia	12	1.34	57	0.95	145	0.99	249	1.00	426	1.16	560	**1.33**	269	**1.63**
	/20	0.75-2.41	/112	0.71-1.27	/287	0.81-1.22	/482	Reference	/751	0.99-1.36	/909	**1.14-1.54**	/395	**1.37-1.94**
Other	2	0.43	22	0.71	62	**0.71**	110	1.00	173	0.89	196	0.89	69	1.03
	/5	0.11-1.76	/36	0.45-1.13	/97	**0.52-0.98**	/144	Reference	/236	0.70-1.13	/274	0.70-1.13	/93	0.76-1.39
**Histology**														
Intestinal	3	**3.31**	8	0.70	27	0.96	65	1.00	145	1.21	201	1.25	109	**1.59**
	/3	**1.01-0.87**	/19	0.34-1.47	/52	0.61-1.51	/125	Reference	/238	0.90-1.62	/330	0.94-1.65	/160	**1.16-2.16**
Diffuse	11	0.88	58	0.81	126	0.81	217	1.00	261	1.05	251	**1.22**	73	**1.73**
	/17	0.48-1.63	/98	0.60-1.08	/217	0.65-1.02	/322	Reference	/381	0.88-1.26	/347	**1.01-1.47**	/92	**1.32-2.27**
Other	5	0.85	34	0.86	129	0.83	279	1.00	459	1.05	538	**1.17**	239	**1.36**
	/10	0.35-2.08	/63	0.6-1.23	/239	0.68-1.03	/451	Reference	/735	0.90-1.22	/811	**1.01-1.35**	/337	**1.14-1.62**
**TNM**														
I	2	**5.51**	1	0.24	5	**0.34**	23	1.00	57	1.45	78	**2.11**	45	**3.01**
	/3	**1.28-3.73**	/23	0.03-1.75	/84	**0.13-0.90**	/137	Reference	/240	0.89-2.36	/237	**1.32-3.36**	/105	**1.82-4.98**

II	2<br>/5	0.96<br>0.24-3.94	14<br>/38	0.77<br>0.44-1.35	35<br>/104	0.71<br>0.48-1.05	90<br>/201	1.00<br>Reference	137<br>/295	1.05<br>0.80-1.37	184<br>/385	1.14<br>0.89-1.47	89<br>/159	**1.48**<br>**1.10-1.99**
III	15<br>/22	0.77<br>0.46-1.30	85<br>/119	0.83<br>0.66-1.05	242<br>/320	0.91<br>0.77-1.06	448<br>/560	1.00<br>Reference	671<br>/819	1.09<br>0.97-1.23	728<br>/866	1.19<br>1.06-1.34	287<br>/325	**1.50**<br>**1.29-1.74**

HR, hazard ratio; CI, confidence interval.

Supplementary Table 2: Stratified analysis of age at diagnosis associated with gastric cancer patients' survival in the period 2003-2004.

Variable	**Age < 30 years**		**Age 30-39 years**		**Age 40-49 years**		**Age 50-59 years**		**Age 60-69 years**		**Age 70-79 years**		**Age≥ 80 years**	
	Deaths /Patients	HR (95% CI)	Deaths /Patients	HR (95% CI)	Deaths /Patients	HR (95% CI)	Deaths /Patients	HR (95% CI)	Deaths /Patients	HR (95% CI)	Deaths /Patients	HR (95% CI)	Deaths /Patients	HR (95% CI)
**Sex**														
Female	5	0.94	26	0.84	104	1.27	143	1.00	196	1.14	278	**1.51**	205	**1.90**
	/12	0.38-2.3	/70	0.55-1.28	/203	0.98-1.63	/338	Reference	/432	0.92-1.42	/515	**1.23-1.85**	/320	**1.53-2.35**
Male	6	0.67	32	0.93	126	0.85	324	1.00	468	1.14	440	**1.39**	229	**1.94**
	/18	0.3-1.51	/77	0.65-1.35	/304	0.69-1.05	/708	Reference	/924	0.99-1.32	/781	**1.2-1.61**	/343	**1.64-2.31**
**Race**														
White	9	0.68	40	0.84	153	0.99	319	1.00	452	1.05	467	**1.28**	292	**1.78**
	/24	0.35-1.33	/96	0.6-1.17	/309	0.82-1.2	/654	Reference	/889	0.91-1.22	/831	**1.11-1.47**	/427	**1.52-2.09**
Black	1	1.66	7	0.94	43	1.34	60	1.00	74	1.31	97	**2.50**	48	**3.01**
	/2	0.23-2.23	/18	0.43-2.07	/85	0.91-1.99	/149	Reference	/156	0.93-1.85	/139	**1.81-3.46**	/62	**2.04-4.42**
Other	1	0.64	11	0.91	34	0.78	88	1.00	138	**1.32**	154	**1.5**	94	**1.84**
	/4	0.09-4.59	/33	0.49-1.72	/113	0.53-1.17	/243	Reference	/311	**1.01-1.72**	/326	**1.15-1.95**	/174	**1.37-2.46**
**Tumor sites**														
Cardia	2	0.60	16	0.73	57	0.8	180	1.00	233	1.05	209	**1.29**	73	**1.99**
	/6	0.15-2.42	/41	0.44-1.23	/125	0.60-1.08	/348	Reference	/429	0.87-1.28	/347	**1.06-1.58**	/99	**1.51-2.62**
Non-cardia	5	0.81	24	0.81	128	1.11	207	1.00	319	**1.24**	396	**1.6**	276	**2.07**
	/15	0.33-1.99	/76	0.53-1.23	/304	0.89-1.38	/542	Reference	/725	**1.04-1.48**	/766	**1.35-1.9**	/451	**1.73-2.48**
Other	4	0.78	18	1.18	45	1.19	80	1.00	112	1.08	113	**1.39**	85	**1.9**
	/9	0.28-2.14	/30	0.71-1.99	/78	0.82-1.72	/156	Reference	/202	0.81-1.44	/183	**1.04-1.85**	/113	**1.39-2.58**
**Histology**														
Intestinal	1	0.55	7	0.85	25	1.07	73	1.00	117	1.06	173	**1.41**	135	**2.07**
	/4	0.08-3.96	/22	0.39-1.85	/67	0.68-1.69	/201	Reference	/310	0.79-1.42	/380	**1.07-1.85**	/227	**1.55-2.75**
Diffuse	6	0.62	36	0.79	129	0.95	186	1.00	229	1.32	182	**1.64**	85	**2.04**
	/16	0.28-1.41	/84	0.55-1.13	/250	0.76-1.19	/369	Reference	/381	1.08-1.6	/277	**1.34-2.02**	/114	**1.57-2.65**
Other	4	1.03	15	0.86	76	0.9	208	1.00	318	1.14	363	**1.53**	214	**2.04**
	/10	0.38-2.78	/41	0.51-1.46	/190	0.69-1.17	/476	Reference	/665	0.96-1.36	/639	**1.29-1.82**	/322	**1.69-2.48**
**TNM**														
I	0	-	1	0.96	3	0.47	13	1.00	50	**2.71**	77	**4.24**	55	**7.44**
	/8		/18	0.13-7.33	/100	0.13-1.66	/211	Reference	/316	**1.47-4.99**	/330	**2.35-7.64**	/149	**4.06-3.63**
II	2	1.28	5	0.51	36	0.89	92	1.00	134	1.13	164	**1.59**	86	**1.88**
	/4	0.31-5.22	/29	0.21-1.25	/114	0.6-1.3	/276	Reference	/364	0.86-1.47	/353	**1.23-2.05**	/161	**1.4-2.53**
III	9	0.68	52	0.71	191	0.99	362	1.00	480	**1.2**	477	**1.57**	293	**1.86**
	/18	0.35-1.32	/100	0.53-0.96	/293	0.83-1.18	/559	Reference	/667	**1.05-1.38**	/613	**1.36-1.8**	/353	**1.59-2.18**

HR, hazard ratio; CI, confidence interval.

Supplementary Table 3: Results of Cox regression and tests of proportional hazards assumption

	Overall		Non-cardia		Cardia	
	Cox Regression (HR ,95% CI)[a]	Therneau–Grambsch tests ρ (χ^2, P)	Cox Regression (HR ,95% CI)[a]	Therneau–Grambsch tests ρ (χ^2, P)	Cox Regression (HR ,95% CI)[a]	Therneau–Grambsch tests ρ (χ^2, P)
**Sex**						
Male vs. Female	1.10 (1.04-1.16)	0.036 (7.62,0.006)	1.02 (0.95-1.09)	0.022 (2.75, 0.097)	1.11 (0.98-1.26)	0.037 (2.26, 0.133)
**Race**						
Black vs. White	0.95 (0.87-1.03)	0.005 (0.17, 0.682)	0.96 (0.87-1.07)	0.008 (0.19, 0.659)	1.11 (0.88-1.41)	-0.001 (0.01, 0.938)
Other vs. White	0.76 (0.71-0.81)	0.030 (5.19, 0.022)	0.82 (0.75-0.89)	0.021 (2.67, 0.102)	0.88 (0.74-1.05)	0.004 (0.02, 0.877)
**Tumor sites**						
Non-cardia vs. Cardia	0.75 (0.70-0.79)	-0.035 (6.86, 0.008)	NA	NA	NA	NA
Other vs. Cardia	1.14 (1.05-1.23)	-0.070 (28.5, <0.001)	NA	NA	NA	NA
**Histology**						
Diffuse vs. Intestinal	1.40 (1.30-1.52)	0.030 (5.29, 0.021)	1.24 (1.12-1.37)	-0.006 (0.13, 0.722)	1.51 (1.28-1.80)	0.025 (1.07, 0.300)
Unknown vs. Intestinal	1.20 (1.11-1.28)	0.012 (0.90, 0.342)	1.15 (1.05-1.26)	0.028 (2.33, 0.127)	1.08 (0.94-1.24)	0.022 (0.79, 0.375)
**TNM**						
Stage III vs. II vs. I	2.61 (2.50-2.73)	0.020 (2.25, 0.134)	2.72 (2.56-2.89)	0.015 (0.74, 0.388)	2.32 (2.15-2.51)	0.043 (3.12, 0.077)

[a] Univariate Cox regression.

NA, Not Applicable.
